# MicroRNA-192 promotes the development of nasopharyngeal carcinoma through targeting RB1 and activating PI3K/AKT pathway

**DOI:** 10.1186/s12957-020-1798-y

**Published:** 2020-02-03

**Authors:** Qingli Huang, Sen Hou, Xiuqing Zhu, Shouzhou Liu

**Affiliations:** 1 Department of Otorhinolaryngology Head and Neck Surgery, Liaocheng People’s Hospital, No.67 Dongchang West Road, Liaocheng, 252000 Shandong Province People’s Republic of China; 2 Department of Otolaryngology, Yanggu People’s Hospital, Liaocheng, Shandong Province People’s Republic of China; 3 Department of Otolaryngology, Chiping People’s Hospital, Liaocheng, Shandong Province People’s Republic of China

**Keywords:** miR-192, Nasopharyngeal carcinoma, RB1, PI3K/AKT pathway

## Abstract

**Background:**

The dysregulation of microRNAs (miRNAs) has been found in diseases and cancers, including microRNA-192 (miR-192). This study was designed to investigate the role of miR-192 in nasopharyngeal carcinoma (NPC) progression.

**Methods:**

The expression levels of miR-192 and some genes were assessed by qRT-PCR and Western blot. The function of miR-192 was investigated through MTT, Transwell, and dual-luciferase reporter assays.

**Results:**

The expression of miR-192 was increased in NPC tissues, and high miR-192 expression predicted poor prognosis in NPC patients. Functionally, upregulation of miR-192 promoted NPC cell migration, invasion, and growth. Furthermore, miR-192 activated EMT and PI3K/AKT pathway to regulate NPC progression. In addition, miR-192 directly targeted RB1 and suppressed its expression in NPC. Moreover, overexpression of RB1 weakened the promoted effect of miR-192 in NPC.

**Conclusion:**

miR-192 promoted cell viability and metastasis in NPC through suppressing RB1 expression and activating PI3K/AKT pathway.

## Background

Nasopharyngeal carcinoma (NPC) refers to malignant tumors occurring on the top and side walls of the nasopharyngeal cavity. The incidence rate in the southern China is higher than that in the northern China, especially in Guangxi, Guangdong, Fujian, and Hunan provinces [1]. People with NPC have a hard time finding it on their own. Even if some symptoms are found in hospitals, 80% of NPC patients are in advanced stages [2]. The cause of NPC mainly involves many factors, including Epstein–Barr virus (EBV) infection, heredity, environment, and eating habits [3]. The treatment of NPC includes radiation therapy, traditional Chinese medicine, surgical treatment, and immunotherapy. Although radiation therapy has increased the 5-year overall survival rate of NPC patients to about 70%, the prognosis is still not optimistic. The main causes of poor prognosis are distant metastasis and recurrence [4]. Therefore, exploring the pathological mechanism of NPC is very necessary to improve therapeutic strategies.

MicroRNAs (miRNAs) are a class of evolutionarily, highly conserved small molecule non-coding RNAs with a length of about 22 nt and a function of post-transcriptional regulation of gene expression [5]. Now, more than 1000 human miRNAs have been discovered. These miRNAs regulate at least 30% of gene expression and participate in a variety of physiological and pathological processes [6]. To date, the dysregulation of some miRNAs has been detected in NPC. For example, miR-34c suppressed tumor growth and metastasis in NPC by targeting MET proto-oncogene (MET) [7]. Additionally, miR-663b promoted tumor cell proliferation, migration, and invasion in NPC through targeting tumor suppressor 2 (TUSC2) [8]. Recently, the different effect of miR-192 has aroused our concern. It has been reported that miR-192 was downregulated in colon cancer, osteosarcoma, and bladder cancer [9–11]. However, upregulation of miR-192 was found in pancreatic ductal adenocarcinoma, neuroblastoma, and gastric cancer [12–14]. It indicted that the abnormal expression of miR-192 has tissue specificity in human cancers. Correspondingly, the function of miR-192 also changed with the type of cancer. Sun et al. reported that miR-192 overexpression suppressed the tumorigenicity of prostate cancer cells by targeting and inhibiting Nin one binding protein (NOB1) [15]. Li et al. found that upregulation of miR-192 promoted the proliferation and metastasis of hepatocellular carcinoma cell by targeting semaphorin 3A (SEMA3A) [16]. It also suggests that the role of miR-192 depends on the type of cancers. Due to the unknown function of miR-192 in NPC, this study was designed to confirm the regulatory mechanism of miR-192 in NPC.

In this study, retinoblastoma 1 (RB1) was predicted to be a target of miR-192. It has been reported that RB1 is a transcriptional regulator and tumor suppressor retinoblastoma protein in cancers [17]. Moreover, overexpression and lack of RB1 were also found to be associated with tumor progression and metastasis in hepatocellular carcinoma [18]. Wang et al. proposed that RB1 inhibited apoptosis during myocyte differentiation [19]. In addition, the interaction between RB1 and miRNAs were also investigated in human cancers. For example, miR-181a promoted growth of thyroid cancer cells by targeting tumor suppressor RB1 [20]. However, the role of RB1 has not been investigated in NPC and needs to be investigated. Besides that, previous studies have shown that PI3K/AKT pathway is involved in pathogenesis of some cancers, such as breast cancer and gastric cancer [21, 22]. However, the effect of miR-192 on PI3K/AKT pathway remains unknown in NPC. Therefore, we investigated whether miR-192 regulates PI3K/AKT pathway in the present study. Moreover, the molecular mechanism of miR-192/RB1 was also analyzed in NPC. This research may provide new insights into its implication in cancer therapeutics.

## Materials and methods

### Experimental sample

Experiment NPC specimens and normal specimens were acquired from 76 patients in Liaocheng People’s Hospital. All the patients were staged following the World Health Organization (WHO) type and the 8th edition of the American Joint Committee on Cancer (AJCC) staging manual. All these specimens were diagnosed by histopathological examination. NPC patients who participated in this study have not received any treatment except for surgery. For prognosis analysis, another 82 paraffin-embedded NPC biopsy tissues were collected from NPC patients with detailed clinical characteristics and long-term follow-up data at Liaocheng People’s Hospital (China) from January 2016 to July 2019. Signed informed consents were obtained from all participating patients and their families prior to tissue sample collection. All experimental protocols were approved by the Institutional Ethics Committee of Liaocheng People’s Hospital (approval number: No. 2017-201) and performed following the World Medical Association Declaration of Helsinki.

### Cell culture and transfection

Human immortalized nasopharyngeal epithelial cell line NP69 and C666-1 NPC cell line were bought from (BeNa Culture Collection, BNCC, Beijing, China). The growth conditions of NP69 and C666-1 cells were 5% CO_2_, 37 °C, and CM2-1 culture solution (90%RPMI-1640 + 10%FBS). miR-192 mimic and inhibitor, RB1 siRNA, and vector (RiboBio, Guangzhou, China) were severally transferred into C666-1 cells. Untreated C666-1 cells were set as the control.

### RT-qPCR

The extraction of total RNA was performed using TRIzol reagent (Invitrogen, Carlsbad, USA). The cDNA solution was synthesized using PrimeScript RT reagent (Takara, Dalian, China). We conducted RT-qPCR using SYBR Green Master Mix II (Takara) on 7500 Fast Real-Time PCR system (ABI, CA, USA). miR-192 or RB1 was normalized to U6 or GAPDH internal reference using the 2^−△△ct^ method. The primers used in our work were as follows: miR-192, forward primer: 5′-GCG GCG GCT GAC CTA TGA ATT G-3′, reverse primer: 5′-ATC CAG TGC AGG GTC CGA GG-3′; U6, forward primer: 5′-CTC GCT TCG GCA GCA CA-3′, reverse primer: 5′-AAC GCT TCA CGA ATT TGC GT-3′; RB1 forward primer: 5′-GAA CAT CGA ATC ATG GAA TCC CT-3′, reverse primer: 5′-AGA GGA CAA GCA GAT TCA AGG TGA T-3′; GAPDH forward: 5′-ACA TCG CTC AGA CAC CAT G-3′, reverse: 5′-TGT AGT TGA GGT CAA TGA AGG G-3′.

### MTT assay

Transfected C666-1 cells (3 × 10^4^ cells/well) were prepared in a 96-well plate. Next, C666-1 cells were severally incubated for 24, 48, 72, or 96 h in fresh medium. After that, 10 μL of MTT solution was added, and cells were continued to be cultured for 4 h. Next, MTT solution was aspirated and Formazan solution was added to fully dissolve the crystals. The absorbance at 490 nm was examined by a microscope (Olympus Corp, Tokyo, Japan).

### Transwell assay

Cell migration and invasion were assessed using Transwell chambers (Corning, Lowell, MA, USA). Transfected C666-1 cells (2 × 10^4^ cells/well) were seeded into the upper chamber of a Transwell insert without (migration) or with (invasion) pre-coated Matrigel. And RPMI-1640 medium containing 10% FBS was added into lower chamber. The cells migrated or invaded through the chambers were fixed using methyl alcohol, stained using crystal violet. Under a microscope (Olympus Corporation, Tokyo, Japan), 5 visual fields were selected for photographing and counting.

### Luciferase reporter assay

The 3′-UTR of wild or mutant RB1 was severally inserted into pcDNA3.1 plasmid vector (Promega, Madison, USA). Next, above plasmid and miR-192 mimic were transfected into C666-1 cells, which were incubated at room temperature for 20 min. After transfection for 48 h, the medium was discarded and washed 1 time with PBS. Finally, we measured the luciferase activity using dual-luciferase assay system (Promega, USA).

### Western blot analysis

RIPA lysis buffer was used to obtain protein samples. Next, 10% SDS-PAGE separated proteins. And protein samples were incubated in PVDF membranes with 5% non-fat milk. Next, protein samples were incubated overnight at 4  °C with RB1, E-cadherin, N-cadherin, Vimentin, PI3K, AKT, p-PI3K, p-AKT, and GAPDH primary antibodies (Abcam, Cambridge, MA, USA). Afterwards, goat polyclonal anti-rabbit IgG secondary antibodies (Abcam, USA) were added to incubate protein samples for 1 h. Finally, ECL (ECL, Pierce) was used to measure protein expression levels. And protein was quantified with the Image Lab Software (Bio-Rad, Kidlington, UK).

### Statistical analysis

Data analyzed by SPSS 18.0 or GraphPad Prism 6 were shown as mean ± SD. Differences between groups were calculated using one-way ANOVA with Bonferroni post hoc test. The Kaplan–Meier method was utilized to determine overall survival rates, and the *P* value was calculated with the long-rank test (*n* = 82). The association between miR-192 and clinical features in NPC patients was calculated through the chi-squared test (*n* = 76). *P* < 0.05 was considered a statistically significant difference.

## Results

### The abnormal expression of miR-192 was detected in NPC tissues using RT-qPCR

**Fig. 1:**
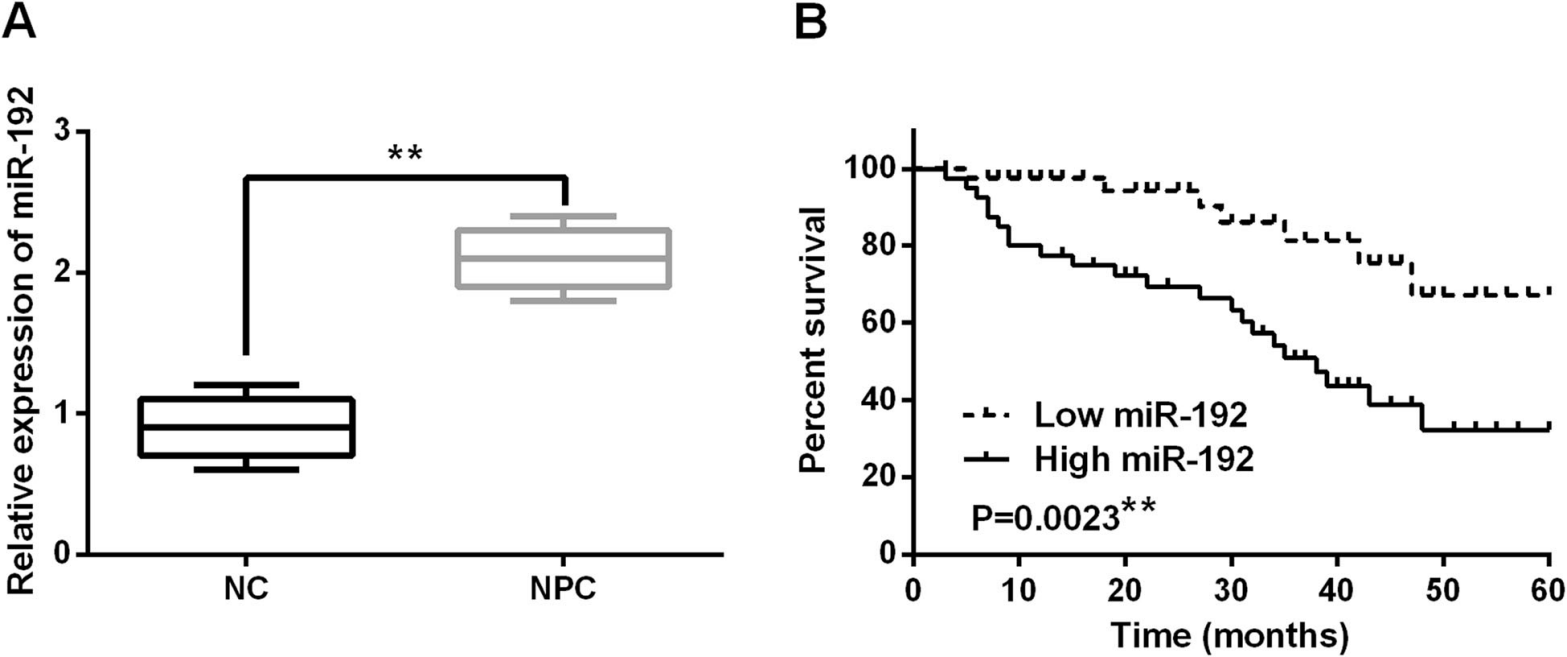
The expression of miR-192 was increased in NPC tissues. **a** miR-192 expressions were identified in NPC tissues and normal tissues (*n* = 76) using RT-qPCR. **b** High expression of miR-192 was correlated with shorter overall survival in NPC patients (*n* = 82). **P < 0.01

miR-192 expression was detected in NPC tissues using RT-qPCR. We found that miR-192 expression was higher in NPC tissues than in normal tissues (*P* < 0.01, Fig. 1a). In addition, high miR-192 expression was closely related to tumor stage or distant metastasis in NPC patients (*P* < 0.05, Table 1). Furthermore, poor prognosis in NPC patients was correlated with high miR-192 expression (*P* < 0.01, Fig. 1b). These results revealed that dysregulation of miR-192 participated in the initiation of NPC.

**Table 1:** Relationship between miR-192 expression and clinic-pathological characteristics of NPC patients

Characteristics	Cases	miR-192		*P* value
		High	Low	
Age (years)				0.47
≥ 60	43	33	10	
< 60	33	27	6	
Gender				0.43
Male	45	32	13	
Female	31	28	3	
WHO type				0.06
I–II	28	18	10	
III	48	42	6	
Distant metastasis				0.03*
Yes	25	20	5	
No	51	40	11	
Tumor stage				0.02*
I–II	23	15	8	
III–IV	53	45	18	

Statistical analyses were performed by the *χ*^2^ test

*P < 0.05 was considered significant

### The effects of miR-192 on cell viability and metastasis were detected in NPC cells using MTT and Transwell assays

**Fig. 2:**
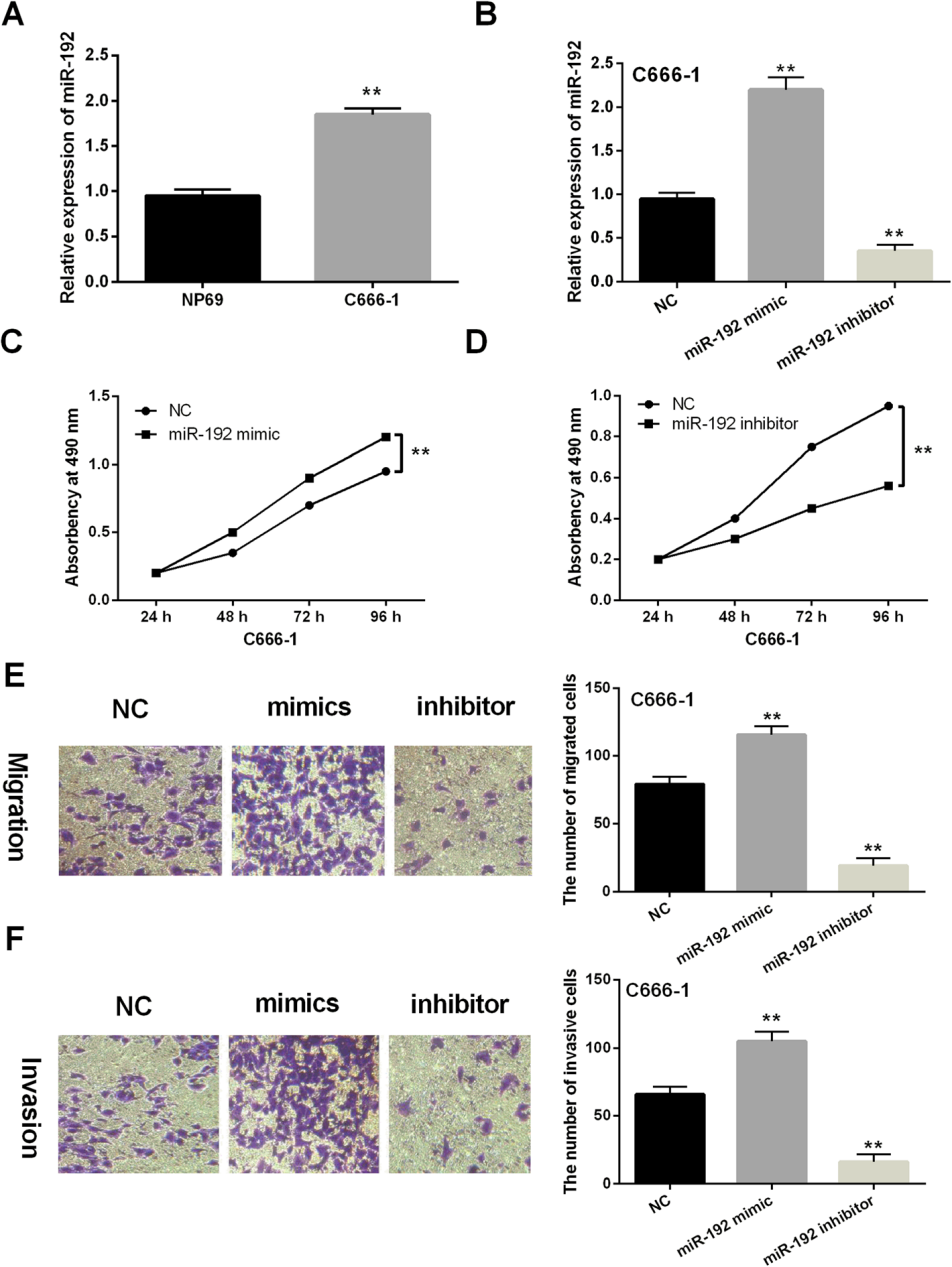
Overexpression of miR-192 promoted cell viability and metastasis in NPC. **a** miR-192 expression was detected in NP69 and C666-1 cell lines using RT-qPCR. **b** miR-192 expression was measured in C666-1 cells with miR-192 mimics or inhibitor using RT-qPCR. **c**–**f** Cell proliferation, migration, and invasion were assessed in C666-1 cells with miR-192 mimics or inhibitor using MTT and Transwell assays. **P < 0.01

Next, miR-192 expression was assessed in NP69 and C666-1 cell lines. Upregulation of miR-192 was identified in C666-1 cells compared to NP69 cells (*P* < 0.01, Fig. 2a). Then, miR-192 mimics or inhibitor was transfected into C666-1 cells to perform gain–loss experiment. miR-192 mimics were found to enhance its expression level, and miR-192 inhibitor decreased its expression (*P* < 0.01, Fig. 2b). Functionally, cell proliferation was promoted by miR-192 mimics and inhibited by its inhibitor in C666-1 cells (*P* < 0.01, Fig. 2c, d). In addition, upregulation of miR-192 was found to promote cell migration. Oppositely, knockdown of miR-192 inhibited cell migration in C666-1 cells (*P* < 0.01, Fig. 2e). Similarly, overexpression of miR-192 promoted cell invasion. Furthermore, cell invasion was suppressed by downregulation of miR-192 in C666-1 cells (*P* < 0.01, Fig. 2f). Collectively, miR-192 promoted cell viability and metastasis in NPC.

### The effect of miR-192 on EMT and PI3K/AKT pathway was investigated in NPC cells using Western blot analysis

**Fig. 3:**
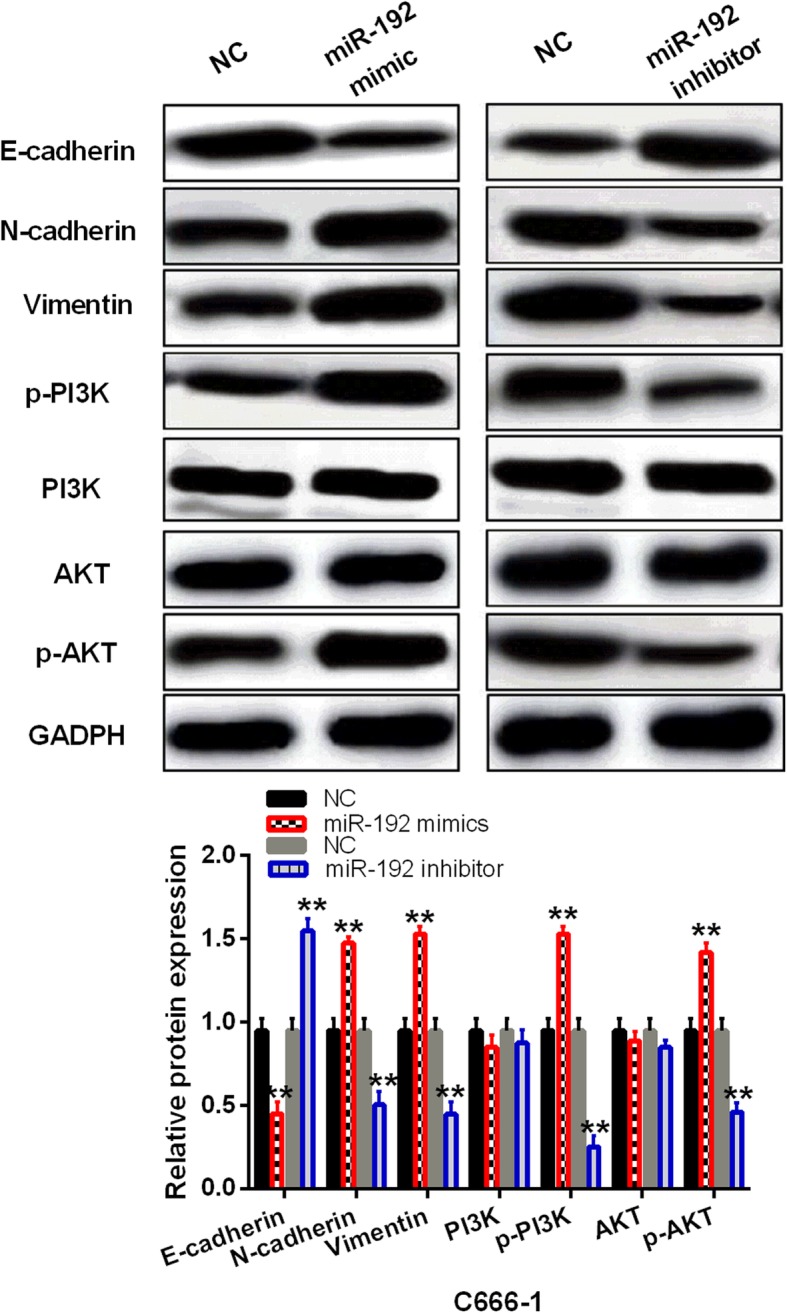
miR-192 activated EMT and PI3K/AKT pathway in NPC. The expressions of E-cadherin, N-cadherin, Vimentin, PI3K, AKT, p-PI3K, and p-AKT were detected in C666-1 cells with miR-192 mimics or inhibitor using Western blot analysis

We also investigated how miR-192 regulates EMT and PI3K/AKT pathway in NPC. We found that upregulation of miR-192 activated EMT through promoting N-cadherin and Vimentin expressions and suppressing E-cadherin (*P* < 0.01, Fig. 3). Inversely, downregulation of miR-192 was found to block EMT (*P* < 0.01, Fig. 3). Besides that, upregulation of miR-192 was found to activate PI3K/AKT pathway in C666-1 cells through promoting p-PI3K and p-AKT expression (*P* < 0.01, Fig. 3). However, knockdown of miR-192 inactivated PI3K/AKT pathway through inhibiting p-PI3K and p-AKT expression (*P* < 0.01, Fig. 3). Therefore, mR-192 regulated NPC progression by activating EMT and PI3K/AKT pathway.

### RB1 was confirmed to be a direct target of miR-192 in NPC cells using luciferase reporter assay

**Fig. 4:**
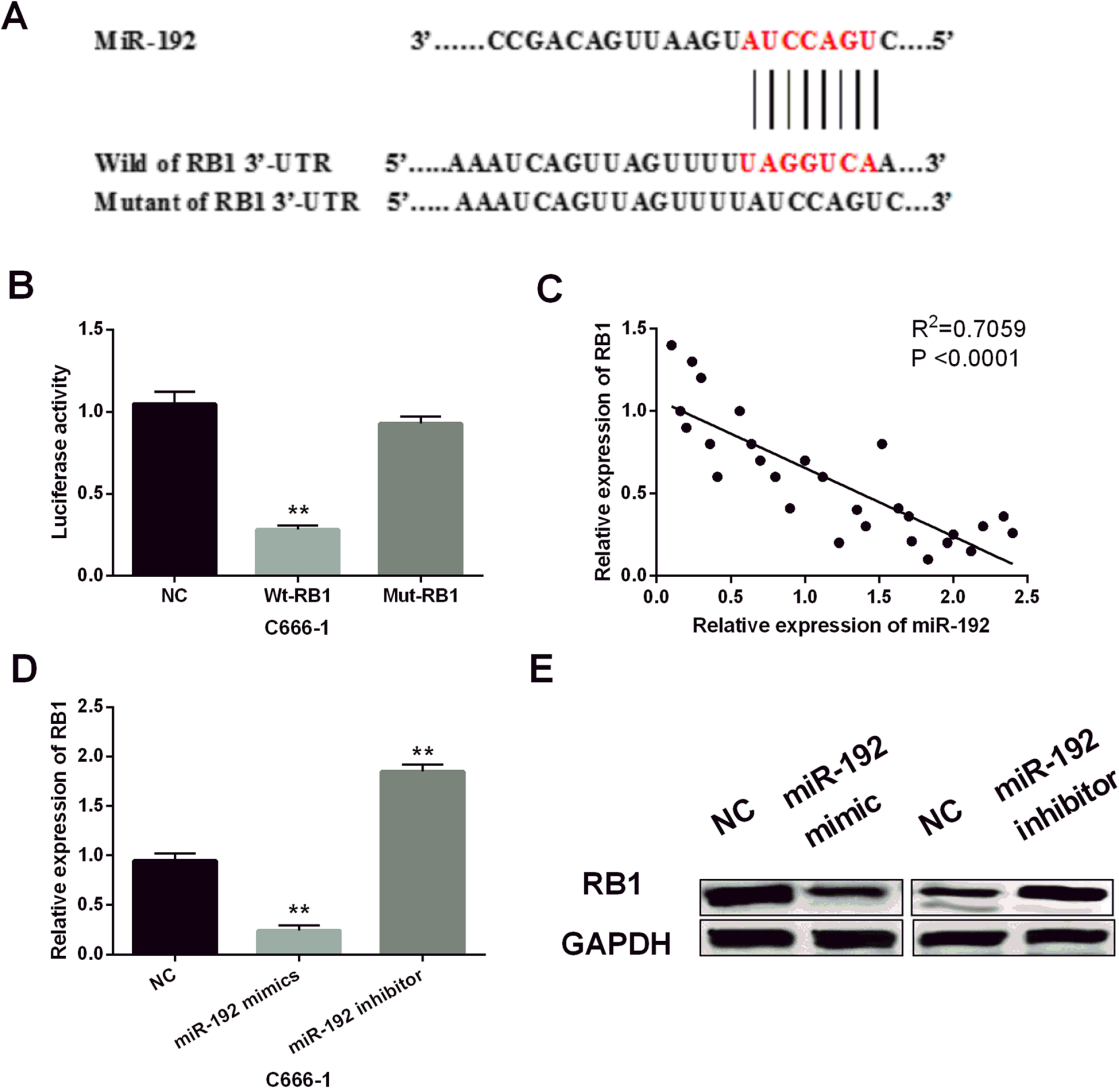
miR-192 regulated RB1 expression in NPC. **a** miR-192 has a binding site with the 3′-UTR of RB1. **b** Dual-luciferase reporter assays were performed to investigate the effects of miR-192 on the 3′-UTR of RB1 activity. **c** The negative correlation between miR-192 and RB1 expressions was found in NPC tissues (*n* = 28) using Spearman correlation analysis. **d**, **e** RB1 expression was detected in C666-1 cells with miR-192 mimics or inhibitor using RT-qPCR and Western blot analysis. **P < 0.01

Further, target genes were searched in TargetScan (http://www.targetscan.org/) to further disclose how miR-192 promotes NPC progression. As shown in Fig. 4a, miR-192 has binding sites with the 3′-UTR of RB1. Luciferase reporter assay suggested that miR-192 obviously reduced the luciferase activity of wild RB1. However, the luciferase activity of mutant RB1 was not influenced by miR-192 (*P* < 0.01, Fig. 4b). Next, we found a negative correlation between miR-192 and RB1 expression in NPC tissues (*P* < 0.01, *R*^2^ = 0.7059; Fig. 4c). After that, RB1 expression in C666-1 cells with miR-192 mimics or inhibitor was measured. Consistent with the above results, miR-192 mimics were found to inhibit RB1 expression, while miR-192 inhibitor promoted RB1 expression (*P* < 0.01, Fig. 4d, e). Hence, miR-192 directly targeted RB1 and suppressed its expression in NPC.

### The interaction between miR-192 and RB1 was found in NPC cells

**Fig. 5:**
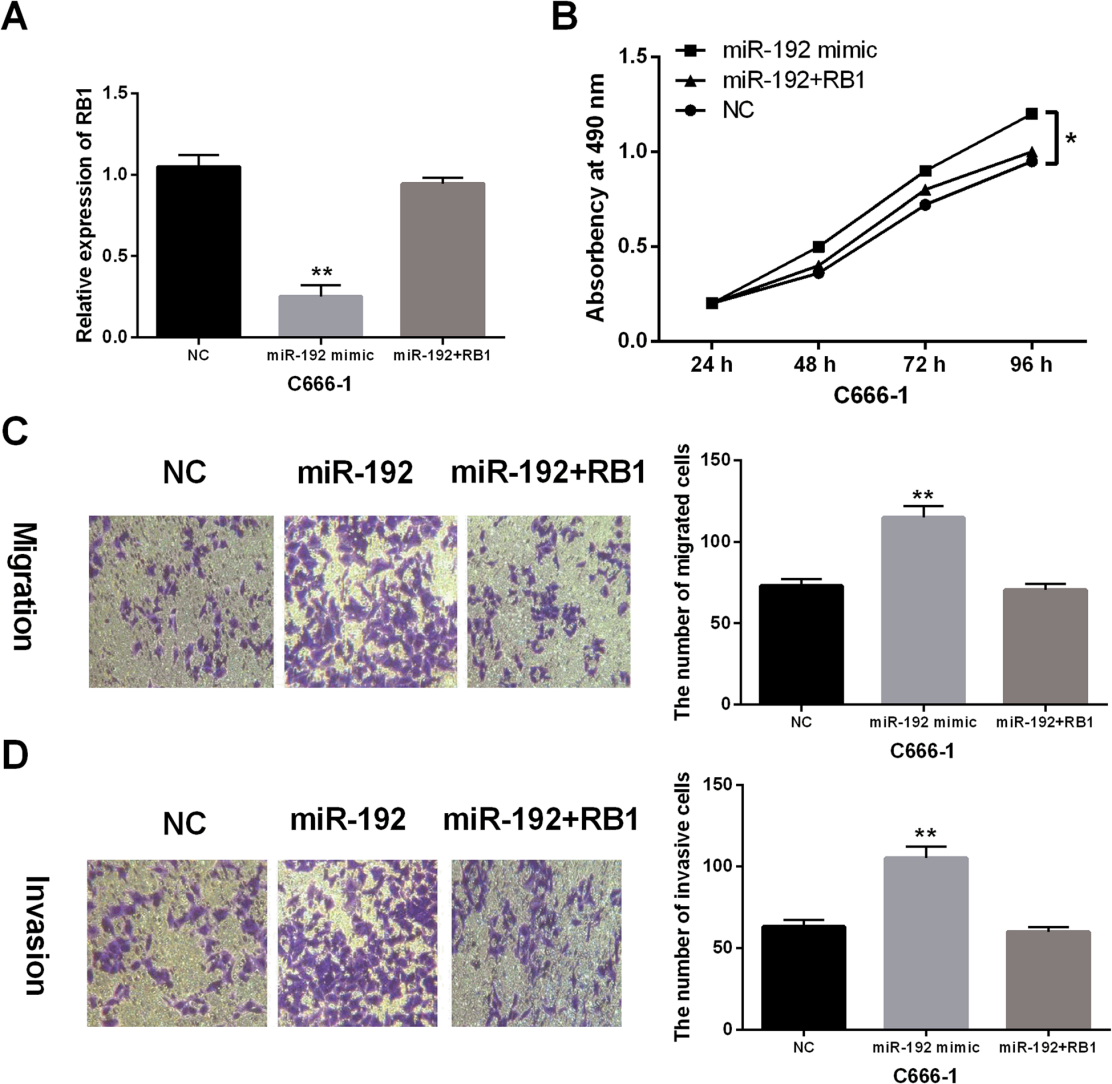
miR-192 promoted the progression of NPC through inhibition of RB1. **a** RB1 expression was detected in C666-1 cells with miR-192 mimics or miR-192 mimics + RB1 vector using RT-qPCR. **b**–**d** Cell proliferation, migration, and invasion were assessed in C666-1 cells with miR-192 mimics or miR-192 mimics + RB1 vector using MTT and Transwell assays. **P < 0.01

In order to explore the interaction between miR-192 and RB1, RB1 vector was transfected into C666-1 cells with miR-192 mimics. First of all, we found that miR-192-induced inhibition of RB1 expression was recovered by RB1 vector in C666-1 cells (*P* < 0.01, Fig. 5a). Functionally, miR-192 mimics promoted cell proliferation in C666-1 cells. But transfection of RB1 vector weakened this increase in C666-1 cell proliferation (*P* < 0.01, Fig. 5b). Meanwhile, miR-192-mediated promotion of cell migration and invasion was also abolished by overexpression of RB1 in NPC (*P* < 0.01, Fig. 5c, d). Taken together, miR-192 exerted promoted effect in NPC through inhibiting RB1 expression.

## Discussion

Many studies have demonstrated that miRNAs take part in the regulation of NPC progression. miR-19a was found to be upregulated and promote development of NPC via targeting TGFβR2 [23]. Similarly, upregulation of miR-192 was also found in esophageal squamous cell carcinoma and squamous cell lung carcinoma [24, 25]. Moreover, miR-192 was upregulated in type 1 diabetes mellitus, regulated pancreatic β cell development, and inhibited insulin secretion through suppressing GLP-1 expression [26]. Furthermore, abnormal expression of miR-192 was related to distant metastasis and prognosis [27, 28]. Functionally, inhibition of miR-192 suppressed human gastric cancer progression [14]. miR-192-5p promoted hepatocellular carcinoma cell proliferation and metastasis through regulating SEMA3A expression [16]. Our research also proposed the upregulation of miR-192 in NPC. Furthermore, high miR-192 expression was related to poor clinical outcome and prognosis in NPC patients. Therefore, we speculated that miR-192 may play a carcinogenic role in NPC.

In order to verify the above speculation, this research was designed. We found that overexpression of miR-192 promoted cell viability and metastasis in NPC. It was consistent with previous studies. Moreover, miR-192 was identified to activate EMT and PI3K/AKT pathway in NPC. Similarly, Zhang et al. found that miR-144 promoted progression of NPC through promoting EMT and PI3K/AKT pathway [29]. In addition, previous studies showed that miR-192 was involved in progression of human cancers through regulating expressions of target genes, such as Egr1 and TCF7 [30, 31]. Here, miR-192 directly targeted RB1 and inhibited its expression in NPC. Furthermore, upregulation of RB1 impaired the promoted effect of miR-192 in NPC.

It has been reported that RB1 functions as a tumor suppressor in the development of human cancers [32]. Moreover, downregulation of RB1 had been detected in breast and bladder cancer [33]. RB1 was found to take part in dysregulation of human cancers, which was regulated by some miRNAs, including miR-26a and miR-335 [34, 35]. Moreover, Zheng et al. revealed that miR-675 promoted glioma cell proliferation and motility by negatively regulating RB1 [36]. In addition, miR-661 promoted tumor invasion and metastasis by activating EMT and directly inhibiting RB1 in non-small cell lung cancer [37]. Consistent with those results, we also identified that miR-192 promoted the development of NPC through suppressing RB1. Besides that, miR-192 was found to exert promoted effect through activating PI3K/AKT pathway in NPC, which has not been investigated in previous studies.

## Conclusion

Briefly, miR-192 was upregulated in NPC, which predicted poor clinical outcome in NPC patients. miR-192 promoted cell viability and metastasis through targeting RB1 and activating PI3K/AKT pathway in NPC. The findings will be beneficial to understand the role of miR-192 in NPC progression.

## Data Availability

The datasets used and/or analyzed during the present study are available from the corresponding author on reasonable request.
